# Hemodynamic factor evaluation using computational fluid dynamics analysis for de novo bleb formation in unruptured intracranial aneurysms

**DOI:** 10.1007/s10072-021-05482-x

**Published:** 2021-07-31

**Authors:** Takehiro Uno, Kouichi Misaki, Kazuya Futami, Iku Nambu, Akifumi Yoshikawa, Tomoya Kamide, Naoyuki Uchiyama, Mitsutoshi Nakada

**Affiliations:** 1 Department of Neurosurgery, Graduate School of Medical Science, Kanazawa University, 13-1 Takara-machi, Kanazawa, Ishikawa 920-8641 Japan; 2 Department of Neurosurgery, Hokuriku Central Hospital, Toyama, Japan

**Keywords:** De novo bleb formation, Computational fluid dynamics, Intracranial aneurysms, Hemodynamic factors, Pressure, Wall shear stress

## Abstract

**Background:**

Although bleb formation increases the risk of rupture of intracranial aneurysms, previous computational fluid dynamic (CFD) studies have been unable to identify robust causative hemodynamic factors, due to the morphological differences of prebleb aneurysm models and a small number of aneurysms with de novo bleb formation. This study investigated the influences of differences in the aneurysm-models and identify causative hemodynamic factors for de novo bleb formation.

**Materials and methods:**

CFD analysis was conducted on three aneurysm models, actual prebleb, postbleb, and virtual prebleb models of two unruptured aneurysms with de novo bleb formation. A new multipoint method was introduced in this study. We evenly distributed points with a 0.5-mm distance on the aneurysm surface of the actual prebleb models (146 and 152 points in the individual aneurysm, respectively), and we statistically compared hemodynamics at the points in the areas with and without bleb formation (19 and 279 points, respectively).

**Results:**

Visually, blebs formed on an aneurysm surface area with similar hemodynamic characteristics in the actual and virtual prebleb models. Statistical analysis using the multipoint method revealed that the de novo bleb formation area was significantly correlated with high pressure (*p* < 0.001), low wall shear stress (WSS) (*p* < 0.001), and the center of divergent WSS vectors (*p* = 0.025).

**Conclusions:**

De novo bleb formation in intracranial aneurysms may occur in areas associated with the combination of high pressure, low WSS, and the center of divergent WSS vectors. The multipoint method is useful for statistical analysis of hemodynamics in a limited number of aneurysms.

## Introduction

Although bleb formation on the aneurysmal wall is associated with an increased risk for rupture of unruptured intracranial aneurysms possibly due to its weakened wall [1–3], mechanisms underlying de novo bleb formation remain unclear. Several studies, using computational fluid dynamics (CFD) analysis, have reported the relationship between de novo bleb formation and hemodynamic factors, including wall shear stress (WSS) [4–7], oscillatory shear index (OSI) [5, 7], and pressure [5, 6]. WSS is defined as the tangential and frictional force exhibited on the aneurysm wall [4–8] by blood flow, and OSI measures the directional change of WSS vectors [9]. However, CFD analysis could not conclusively identify robust causative hemodynamic factors due to the results differing to other studies. In previous reports, CFD analysis was performed using two models: an actual prebleb model and a virtual prebleb model. The former is created from the three-dimensional (3D) imaging data obtained from aneurysms before de novo bleb formation [6, 7], and the latter is created by manually removing blebs from the imaging data of aneurysms after de novo bleb formation [4, 5]. The morphology of the aneurysmal dome may change during a clinical observation period or in conjunction with de novo bleb formation [10]. This may result in morphological differences in an aneurysmal dome between real human aneurysms just before de novo bleb formation and the actual and virtual prebleb models and can cause misleading findings in CFD. In addition, it is extremely rare to obtain 3D images to create an actual prebleb model of aneurysms with de novo bleb formation, making it difficult to statistically compare hemodynamic factors of aneurysms with and without de novo bleb formation.

This study presents two unruptured intracranial aneurysms that exhibited de novo bleb formation during clinical observations. We succeeded in obtaining 3D imaging data of aneurysms both, before and after de novo bleb formation, which enabled hemodynamic evaluation by using CFD analysis of the actual and virtual prebleb models. Moreover, we introduced a new multipoint method to statistically compare hemodynamics at points in areas with and without de novo bleb formation. The purpose of this study was to identify hemodynamic factors associated with de novo bleb formation by statistically comparing hemodynamics using the multipoint method in addition to the visual inspection of the results of CFD analysis for actual and virtual prebleb models. By identifying the true causative hemodynamic factors for de novo bleb formation, it is possible to detect aneurysms that are prone to de novo bleb formation in the future. In other words, this will lead to the identification of aneurysms that are prone to rupture and will help in considering the indications for treatment of unruptured intracranial aneurysms.

## Material and methods

### Case presentations

Two men aged 60 s and 70 s years (Case 1 and 2, respectively) underwent a medical check-up of the brain, and unruptured intracranial aneurysms were identified. The aneurysm in Case 1 was 3.5 mm in diameter and located on the middle cerebral artery (MCA) bifurcation. For Case 2, the aneurysm was 3.8 mm in diameter and located on the anterior communicating artery (AcomA) with a dominant left A1 segment of the anterior cerebral artery. Due to the aneurysms being small and asymptomatic, they were elected to undergo follow-up with annual three-dimensional (3D) computed tomographic angiography (CTA). During the follow-up period, the aneurysms in Cases 1 and 2 had de novo bleb formation 1 and 2 years later, respectively. The bleb is defined as a small, definite protrusion on the aneurysm surface. This study was approved by the institutional review board, and all patients provided informed consent prior to the study.

### Aneurysm modeling

**Fig. 1:**
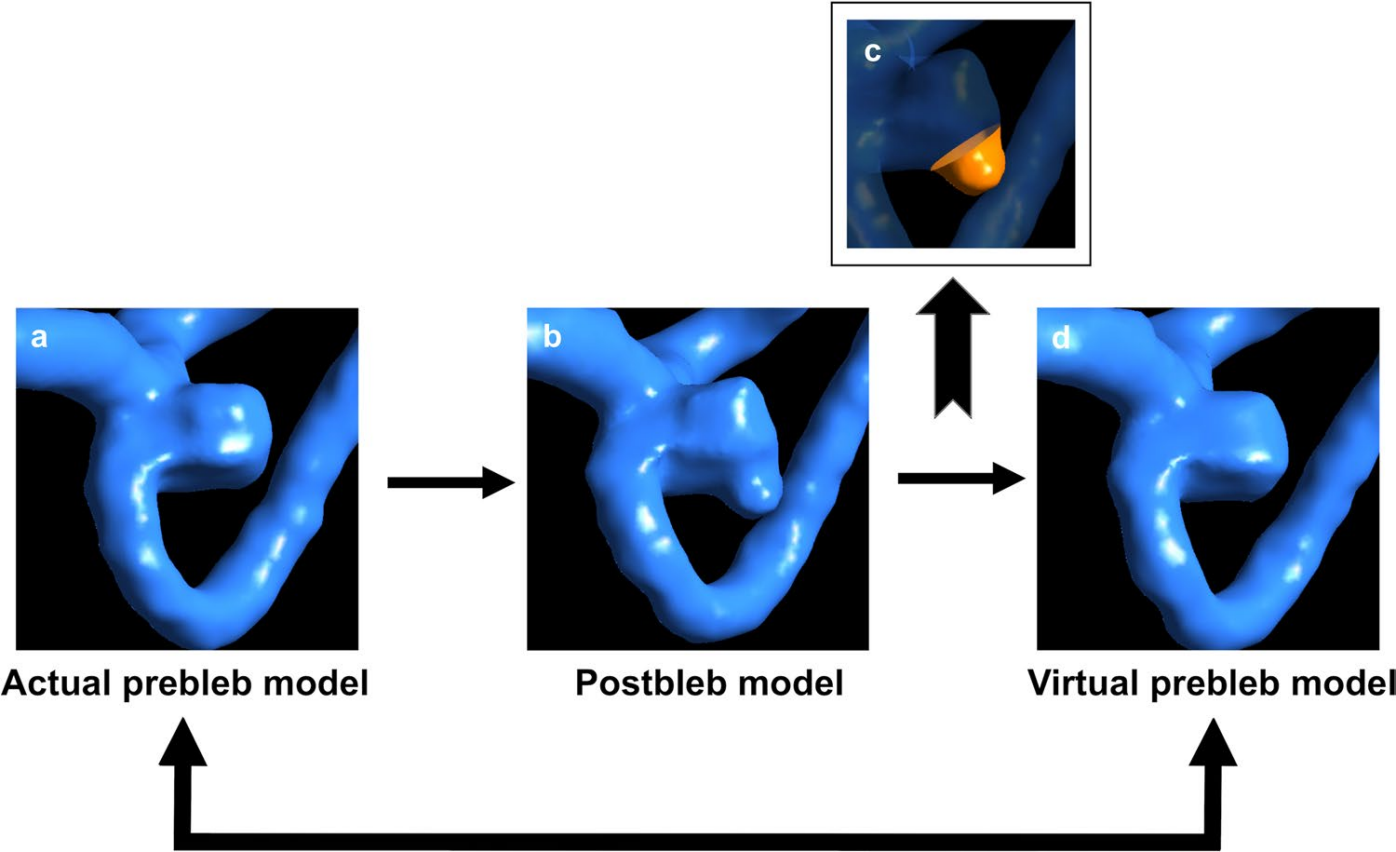
Three vessel models created for an aneurysm in Case 1. **a** The actual prebleb model created from image data obtained by three-dimensional computed tomographic angiography (3D CTA) before de novo bleb formation. **b** Postbleb model based on 3D CTA image data after de novo bleb formation. **d** The virtual prebleb model created by manually removing bleb (**c**) from postbleb model (**b**)

3D CTA was performed using a multislice CT scan system (Aquilion multi 16, Toshiba Medical Systems, Otawara, Japan). Contrast-enhanced CTA utilized the following scan parameters: 0.75-s rotation; scanning pitch, 0.69; 120 kVp, 300 mA; and voxel size, 0.21 × 0.21 × 0.50 mm. An iodinated contrast agent (80–100 mL; Omnipark 300 Daiichi-Sankyo, Tokyo, Japan) was injected at a rate of 3.5 ml/s without saline solution. Following the contrast agent reaching the internal carotid artery, approximately 15 s after injection, the imaging commenced. Blood vessels were extracted and converted into standard triangulated surfaces using Amira version 5.6 software (Maxnet Co, Ltd, Tokyo, Japan). These 3D images were imported into ICEM CFD version 16.2 software (ANSYS Inc., Canonsburg, PA, USA) to assess vessel structure. For each aneurysm, the actual prebleb and postbleb models were created from 3D imaging data of aneurysms before and after de novo bleb formation, respectively. The virtual prebleb model was created by manually removing the bleb to smooth the wall surface of the aneurysmal dome (Fig. 1) on the basis of the consensus of three neurosurgeons (KM, KF, and IN).

### Numerical simulations

The fluid domains of blood vessel models were created using the ANSYS ICEM CFD software, to create meshes comprising tetrahedrons and seven prism element layers near the surface wall in the boundary [11]. A 75-mm passage was added to the proximal plane of the vessel structure to generate a sufficient the inlet length [12]. The density and dynamic viscosity of blood were defined as 1100 kg/m^3^ and 0.0036 Pa, respectively, and were modeled as a Newtonian fluid. The wall of the blood vessel was defined as a rigid no-slip boundary condition. ANSYS CFX version 16.2 (ANSYS Inc.) was used to solve the pulsatile-flow governing Navier–Stokes equations [13–15]. The flow-rate waveform at the inlet was set to be 1.80 s, according to a report by Ford et al. [16]. Zero pressure was imposed at the outlets, and the boundary conditions applied to all models were the same [17]. The interval time for calculation was set to 0.005 s. Two cardiac cycles were simulated, and the results from the peak systole of the second cycle were used in the analysis [18].

### Data analysis

The results of CFD analysis were visualized so that the aneurysm wall information and blood flow state in the aneurysm could be evaluated. The structure of the major intra-arterial flows was determined using flow velocity maps drawn on the cutting surface. Contour maps of hemodynamic parameters, including normalized pressure, normalized WSS, time-averaged wall shear stress (TAWSS) and OSI, and WSS vectors, were exhibited on the aneurysmal wall in vessel models. The pressure and WSS were normalized to the parent vessel values, generated from the same CFD simulation, to minimize dependency on the inlet conditions. For normalization, each value was divided by the average value on the inlet plane, 1 mm proximal to the aneurysm. The normalized analysis values were calculated using the following equation:

$$\mathrm{Normalized}\;\mathrm{Pressure}=\frac{\mathrm{Pressure}}{\mathrm{PressureAVE}(\mathrm{inlet})},\;\mathrm{normalized}\;\mathrm{WSS}=\frac{\mathrm{WSS}}{\mathrm{WSSAVE}(\mathrm{inlet})}$$

In this equation, PressureAVE (inlet) and WSSAVE (inlet) indicate the average pressure and the average WSS at the inlet plane, respectively. The relationship between the de novo bleb formation area and contour maps of the hemodynamic parameters and WSS vectors on the aneurysm wall were visually evaluated. In the CFD analysis of the actual and virtual prebleb models, we visually characterized hemodynamic features of the de novo bleb formation area by comparing them with those in the area without de novo bleb formation. Contour maps of the hemodynamic parameters and WSS vectors on the aneurysm wall were visually evaluated. According to the bleb location in the postbleb model, the de novo bleb formation area in the actual prebleb model was determined based on the consensus of three neurosurgeons (KM, KF, and IN).

**Fig. 2:**
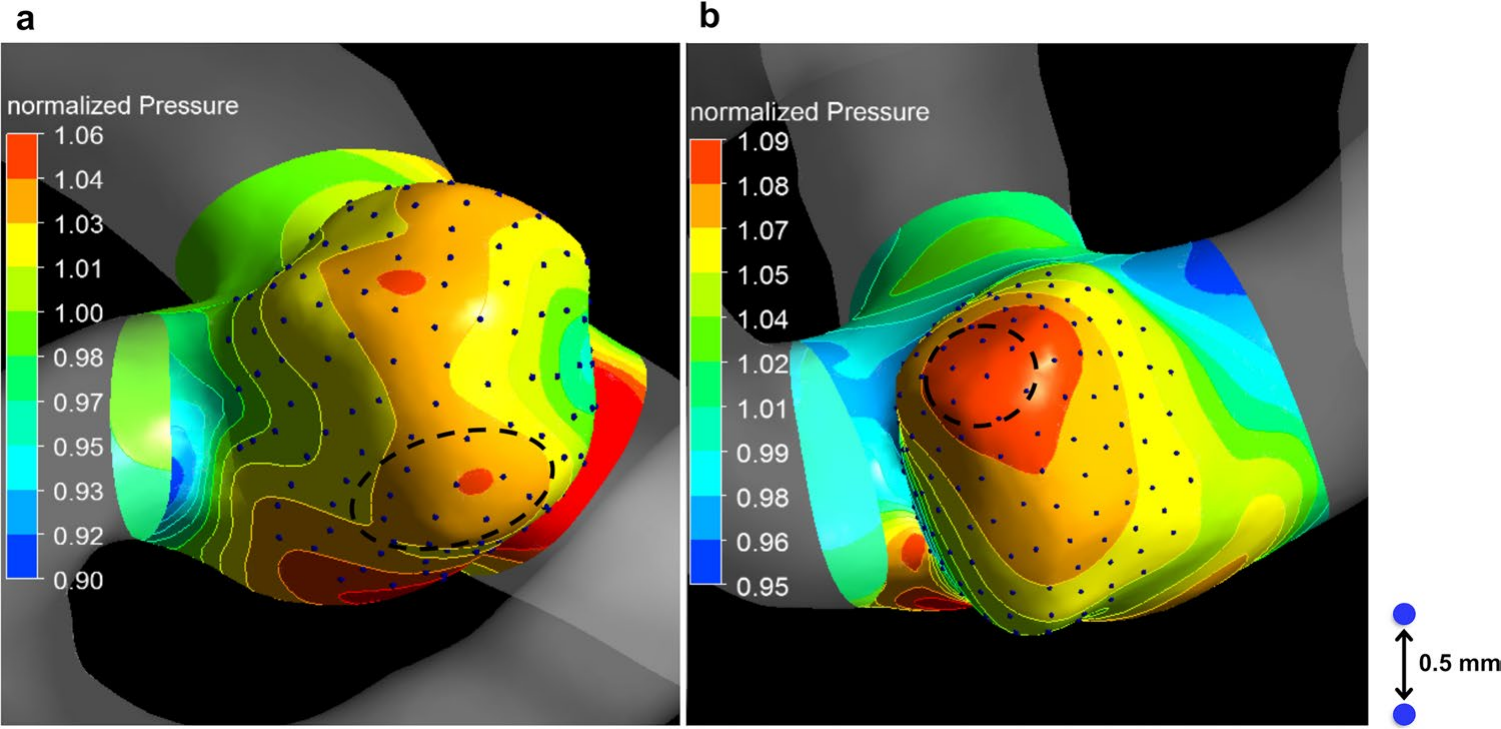
Points evenly distributed with a 0.5-mm distance using an ANSYS CFX function of the point cloud on the whole aneurysm surface, which exhibits the contour maps of normalized pressure in the actual prebleb models of Cases 1 (**a**) and 2 (**b**). Hemodynamic parameters were measured at the center of a boll mark which was the symbol of the distributed points. The dotted circle indicates the area of de novo bleb formation. Statistical analysis was performed to compare hemodynamics at points on areas with and without de novo bleb formation

Using the ANSYS CFX function of the point cloud, we evenly distributed points with a 0.5-mm distance on the surface of the whole aneurysm of the actual prebleb model (Fig. 2). Consequently, the number of distributed points was 146 (11 points in the de novo bleb formation area and 135 in the area without de novo bleb formation) in Case 1 (Fig. 2a) and 152 (8 points in the de novo bleb formation area and 135 in the area without de novo bleb formation) in Case 2 (Fig. 2b). Hemodynamic parameters were measured at the center of a boll mark which was the symbol of the distributed points (Fig. 2). The centers of divergent WSS vectors were 2 (1 in each area with and without de novo bleb formation) in both Cases 1 and 2. We statistically compared the hemodynamic values and the number of centers of divergent WSS vectors at points in the areas with and without de novo bleb formation.

### Statistical analysis

Continuous data were reported as mean ± standard deviation for continuous variables. The Mann–Whitney *U* test and Fisher’ s exact test were used to analyze parameters, as appropriate. Statistical significance was indicated when *p* < 0.05. Statistical analysis was performed using SPSS (IBM SPSS Statistics 24, Chicago, IL, USA).

## Results

**Fig. 3:**
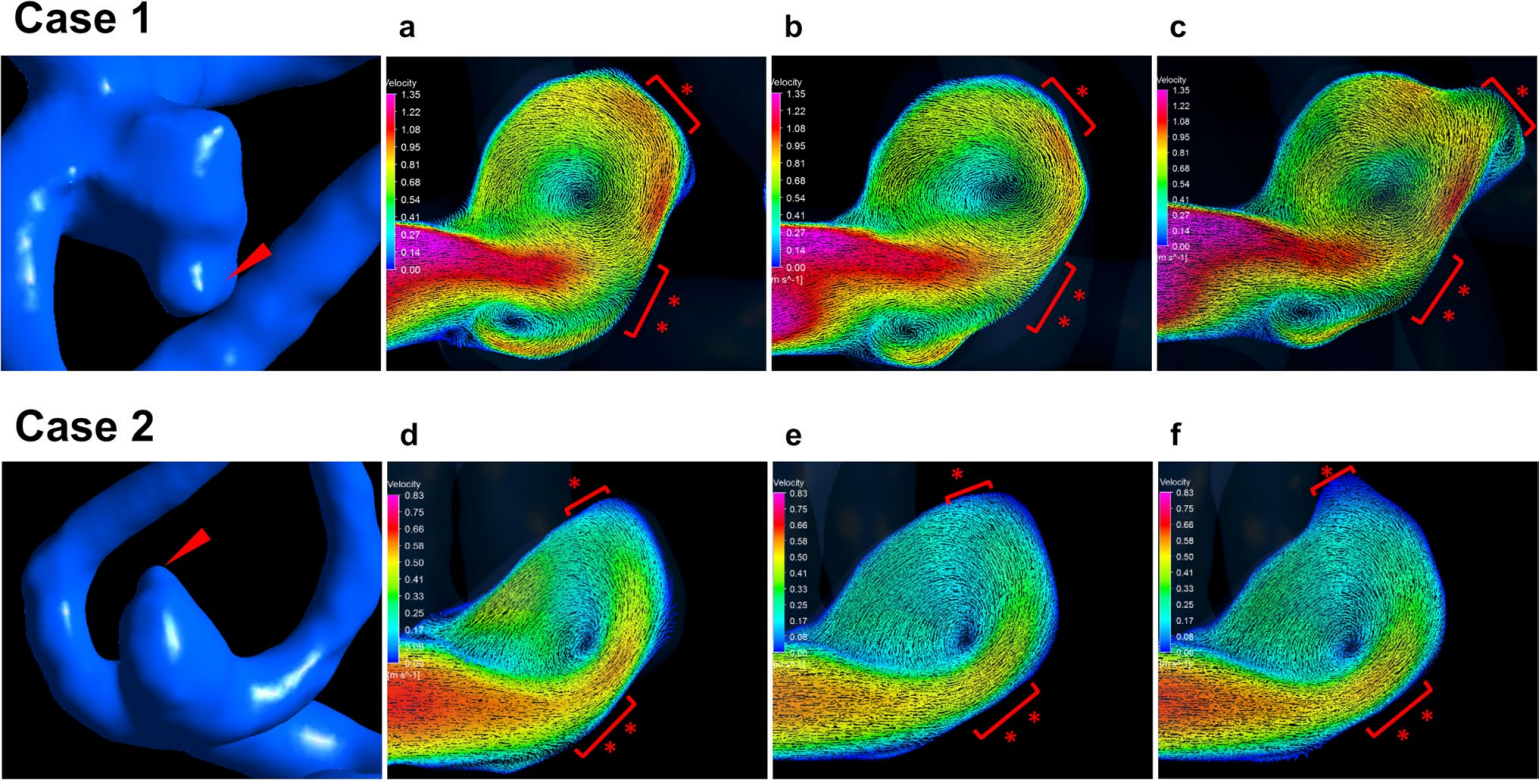
A right middle cerebral artery aneurysm in case 1 (upper row) and anterior communicating artery aneurysm in case 2 (lower row). Red arrowheads show bleb lesions in both cases. **a**–**f** Blood flow velocity maps at peak systole in the actual prebleb (**a** and **d**), virtual prebleb (**b** and **e**), and postbleb (**c** and **f**) models. In both cases, the bleb occurred around the area (*) where the high flow vectors changed the flow direction accompanied by a decrease in the flow velocity rather than around the inflow impingement zone (**)

High-velocity flow vectors and the location of de novo bleb formation in the actual prebleb, virtual prebleb, and postbleb models in Cases 1 and 2 (Fig. 3a–c and d–f) are shown in Fig. 3. The bleb occurred in the area where the high flow vectors changed the flow direction accompanied by a decrease in the flow velocity rather than around the inflow impingement zone.

**Fig. 4:**
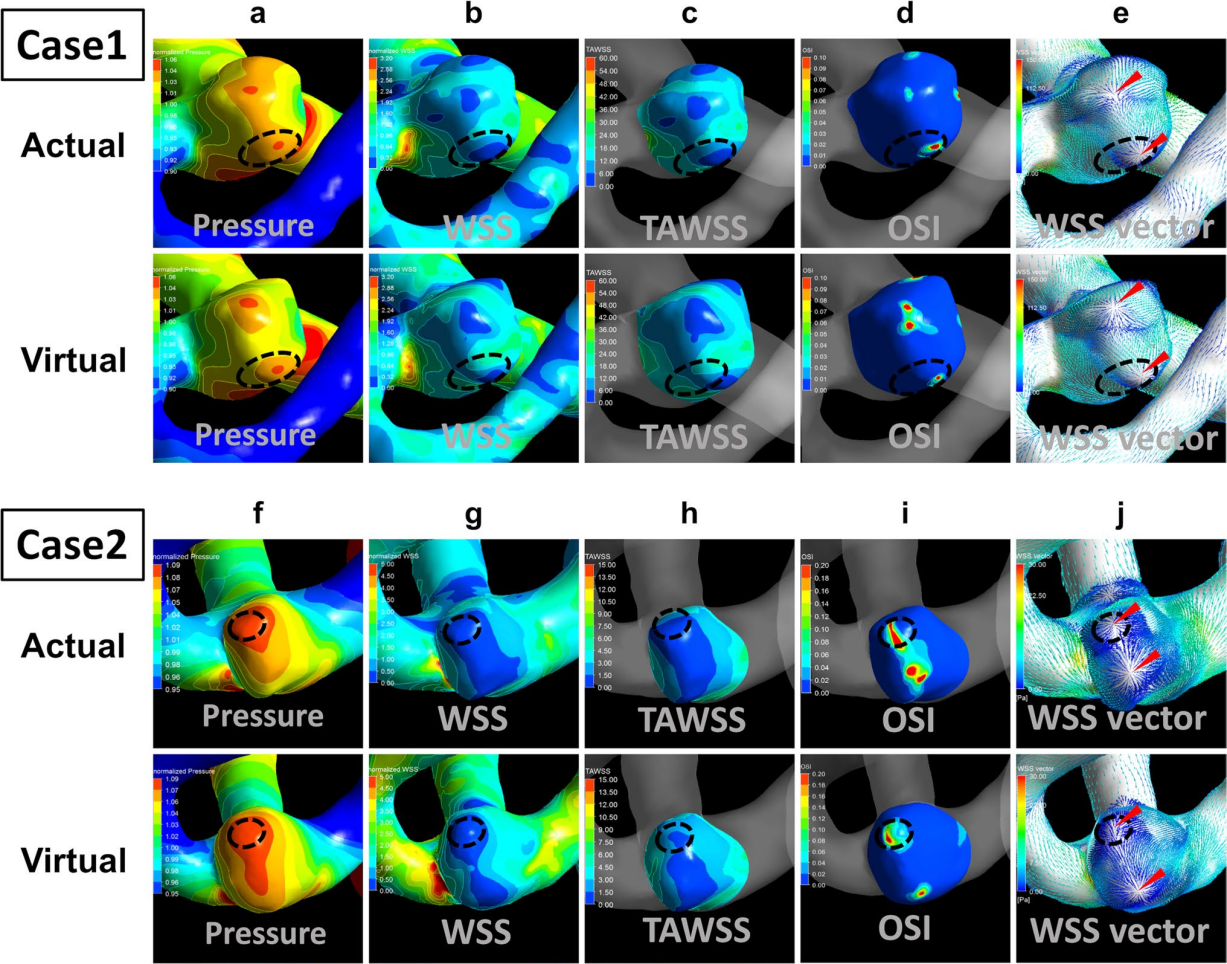
Contour maps of normalized pressure (**a** and **f**), normalized wall shear stress (WSS) (**b** and **g**), time-averaged wall shear stress (TAWSS) (**c** and **h**), and oscillatory shear index (OSI) (**d** and **i**) in addition to WSS vectors (**e** and **j**) on the aneurysm wall in the actual and virtual prebleb models for an aneurysm in both cases. The results of normalized pressure, normalized WSS, and WSS vectors were used at the peak systole of the second cycle. The dotted line indicates the area of de novo bleb formation, and arrowheads indicate the center of divergent WSS vectors. Computational fluid dynamics analysis revealed that the bleb occurred in the region of high normalized pressure, low normalized WSS, low TAWSS and partially high OSI, and the center of divergent WSS vectors in both the actual and virtual prebleb models

Contour maps of normalized pressure; normalized WSS, TAWSS, and OSI; and WSS vectors in the actual and virtual prebleb models in Cases 1 and 2 are shown in Fig. 4. Similarly, in both cases a bleb occurred in the region of high normalized pressure, low normalized WSS, low TAWSS, and partially high OSI in both actual and virtual prebleb models (Fig. 4a–d and f–i). Each aneurysm had two centers of radically divergent WSS vectors. The de novo bleb formation area included the center in both the actual and virtual prebleb models of Cases 1 and 2 (Fig. 4 e and j).

Statistical analysis using the multipoint method revealed that points in the de novo bleb formation area showed significantly higher normalized pressure (*p* < 0.001, < 0.001, and < 0.001) and lower normalized WSS (*p* < 0.001, = 0.033, and < 0.001) in Case 1, Case 2, and total, respectively, compared to the area without de novo bleb formation (Table 1). The points with maximum normalized pressure (1.04 and 1.09 in Case 1 and Case 2, respectively) and with minimum normalized WSS (0.11 and 0.02 in Case 1 and Case 2, respectively) existed in the de novo bleb formation area of both aneurysms. No significant differences were observed in the areas with and without de novo bleb formation in TAWSS (*p* = 0.54, = 0.71, and = 0.14 in Case 1, Case 2, and total, respectively) and OSI (*p* = 0.70, = 0.55, and = 0.53 in Case 1, Case 2, and total, respectively). The number of centers of divergent WSS vectors was 2 in the de novo bleb formation area, where 19 points were evenly distributed and 2 in the area without de novo bleb formation, where 279 points were evenly distributed (*p* = 0.25).

**Table 1:** The magnitude of hemodynamic parameters of points in the areas with and without bleb formation

	Case 1 (*n* = 146)			Case 2 (*n* = 152)			Total (*n* = 298)		
	Bleb formation area (*n* = 11)	Area without bleb formation (*n* = 135)	*P* value	Bleb formation area (*n* = 8)	Area without bleb formation (*n* = 144)	*P* value	Bleb formation area (*n* = 19)	Area without bleb formation (*n* = 279)	*P* value
Normalized Pressure	1.04 ± 0.005	1.01 ± 0.05	< 0.001*	1.09 ± 0.005	1.05 ± 0.03	< 0.001*	1.06 ± 0.03	1.03 ± 0.04	< 0.001*
Normalized WSS	0.33 ± 0.17	0.76 ± 0.30	< 0.001*	0.52 ± 0.32	0.93 ± 0.53	0.033*	0.42 ± 0.30	0.85 ± 0.44	< 0.001*
TAWSS (Pa)	7.28 ± 3.09	12.61 ± 5.99	0.054	1.33 ± 0.72	2.81 ± 1.64	0.071	4.30 ± 3.72	7.43 ± 6.49	0.14
OSI	0.0046 ± 0.0044	0.0030 ± 0.0024	0.70	0.0145 ± 0.0125	0.0113 ± 0.0269	0.55	0.0095 ± 0.0106	0.0073 ± 0.0199	0.53
The center of divergent WSS vectors, N (%)	1 (9.0)	1 (0.7)	0.156	1 (12.5)	1 (0.7)	0.114	2 (10.5)	2 (0.7)	0.025*

Values are shown as mean ± SD when appropriate. Mann–Whitney *U* test was used for statistical analysis of all parameters except for the center of divergent WSS vectors for which Fisher exact test was used. A *P* value < 0.05 was considered significant. * indicates significant; *WSS* wall shear stress; *TAWSS* time-averaged wall shear stress; *OSI* oscillatory shear index; *n* the number of points evenly distributed on the aneurysm surface; *N* the number of the center of divergent WSS vectors

## Discussion

In two cases of an unruptured MCA or AcomA aneurysm with de novo bleb formation during the observation period, we performed hemodynamic evaluation using CFD analysis for actual and virtual prebleb models, reconstructed from 3D imaging data of aneurysms both, before and after de novo bleb formation, respectively. Additionally, we first introduced a multipoint method to enable statistical analysis to compare hemodynamics of the aneurysm wall in a small number of aneurysms with de novo bleb formation.

Table 2 lists previous reports describing hemodynamic parameters associated with de novo bleb formation in unruptured intracranial aneurysms evaluated using CFD analysis [5–7]. We excluded growing blebs or aneurysmal domes, which did not match the criteria for de novo bleb formation. Studies that did not include a discriminated cohort of unruptured aneurysms were excluded, because a rupture point of a ruptured aneurysms may change into a bleb-like form immediately after rupturing [19, 20]. These studies were unsuitable for investigating the mechanisms of de novo bleb formation on unruptured aneurysmal walls. Consequently, only 3 previous reports matched the criteria of this study (Table 2).

**Table 2:** Hemodynamic parameters associated with de novo bleb formation in unruptured intracranial aneurysms

	Unruptured aneurysms (n)	Model for CFD	Pressure	WSS	OSI	WSS vector	Relation with intra-aneurysm flow
Russell et al. (2013) [5]^a^	9	Virtual	-	Maximum (78%)	-	-	-
Sugiyama et al. (2016) [6]	1	Actual	High	Heterogenous	-	-	Along low velocity flow after inflow impingement
Machi et al. (2017) [7]	3	Actual	-	Low	High	-	-
Present case (2020)	2	Actual and Virtual	High	Low	High	Divergent	Along major intra-aneurysmal flow

^a^Within 9 unruptured aneurysms of total 27, the relationship of bleb formation and pressure, OSI, WSS vector, or intra-aneurysmal flow was not examined. *n* the number of aneurysms; *CFD* computational fluid dynamics; *WSS* wall shear stress; *OSI* oscillatory shear index; -, not examined

Previous studies using CFD analysis to investigate mechanisms underlying de novo bleb formation could not identify robust causative hemodynamic factors because the results differed from those of other studies. Cebral et al. [4] evaluated 20 aneurysms with 30 blebs by using CFD analysis for virtual prebleb models and showed that 60% of blebs occurred around the inflow impingement zone and that 80% of blebs occurred around the region with the highest WSS values in the aneurysmal wall. However, we excluded their study from Table 2 as they did not show hemodynamic characteristics of a cohort of unruptured aneurysms with de novo bleb formation. Russel et al. [5] showed that seven blebs (78%) of 9 unruptured aneurysms with single de novo bleb formation occurred around the maximum WSS point of aneurysmal walls in virtual prebleb models. However, their study did not evaluate the relationship between low WSS and de novo bleb formation area [5]. Conversely, Sugiyama et al. [6] showed that de novo bleb formation occurred in a region with low velocity just after inflow impingement and with heterogeneously distributed WSS on CFD analysis for an actual prebleb model. Machi et al. [7] performed CFD analysis for actual prebleb models and reported that the de novo bleb formation area showed low WSS. In the present study, de novo bleb formation occurred around the area where the major intra-aneurysmal flow changed the flow direction accompanied by a decrease in the flow velocity rather than around the flow impingement zone. Moreover, de novo bleb formation arose from the low WSS area, including the minimum WSS point on the aneurysmal wall in both the actual and virtual prebleb models. In 15 unruptured aneurysms with de novo bleb formation reported in previous studies and the present study, the de novo bleb formation area was associated with high WSS in 7, low WSS in 5, heterogenous in 1, and unknown in 2. This conflicting result may be attributed to the uncertainty of the role of WSS in de novo bleb formation or the heterogeneity of mechanisms underlying de novo bleb formation.

Few studies have reported the role of OSI in de novo bleb formation [5, 7]. Russel et al. [5] first investigated OSI on aneurysmal walls with de novo bleb formation and reported that maximal OSI was not significantly related to de novo bleb formation. However, they did not study the role of OSI in de novo bleb formation in a cohort of unruptured aneurysms. Machi et al. [7] showed that de novo bleb formation was related to high OSI coupled with low WSS. Regarding the relationship between pressure and de novo bleb formation, Sugiyama et al. [6] demonstrated that high pressure on the aneurysmal wall was associated with de novo bleb formation. In the current study, de novo bleb formation occurred in the region of high normalized pressure and partially high OSI in both actual and virtual prebleb models. In fact, Sugiyama et al. [6] observed intra-operatively a thin and reddish wall of the daughter sac, which was exposed to high pressure on CFD analysis. It is likely that high pressure causes thinning of the aneurysmal wall, leading to de novo bleb formation.

In the present study, we investigated the features of WSS vectors with respect to de novo bleb formation, which had not been previously examined. As a result, WSS vectors had radically divergent directions in the de novo bleb formation area, and statistically, the de novo bleb formation area in our cases significantly included the center of divergent WSS vectors (*p* = 0.025). Using CFD analysis, Senko et al. [21] evaluated hemodynamics on the aneurysm wall that ruptured during the surgical procedure and reported that the ruptured dome exhibited high pressure and divergent WSS vectors. Kim et al. [22] examined the relationship between thin-walled regions of aneurysm surfaces and hemodynamic parameters, including newly employed WSS divergence, which is a hemodynamic parameter accounting for both magnitude and directionality of WSS. They reported that the highest quartiles of pressure and WSS divergence corresponded with 73.3% and 86.7% of thin-walled regions of unruptured aneurysms, respectively. The excessive wall tension caused by high pressure and divergent WSS vectors may increase the fragility of the aneurysmal wall and may lead to de novo bleb formation.

Although the virtual prebleb model is created from the 3D imaging data of aneurysms with blebs, the morphology of the aneurysmal dome may change during the observation period after de novo bleb formation or in conjunction with de novo bleb formation [10]. Conversely, the actual prebleb model is created from 3D imaging data of aneurysms before de novo bleb formation. The morphology of the aneurysmal dome just before de novo bleb formation may differ from that of the aneurysm that provided the 3D imaging data for the actual prebleb model. The change in the morphology of an aneurysmal dome during a clinical observation period may cause misleading of the CFD analyses and conflicting results. This indicates the requirement to validate both the actual and virtual prebleb models. In the present study, we validated the prebleb models by comparing the results of CFD analysis between actual and virtual prebleb models. CFD analyses for both actual and virtual prebleb models showed the same results. This finding suggests that the conflicting results of previous studies using CFD analysis may not be attributed to the morphological differences of the prebleb models.

Because de novo bleb formation in unruptured aneurysms is extremely rare, it is difficult to obtain a sufficient number of actual prebleb models for statistical analysis of the mechanisms of de novo bleb formation. In the present study, we introduced a multipoint method to statistically compare hemodynamics on the aneurysm wall areas with and without de novo bleb formation. In this study, the de novo bleb formation area showed the combination of significantly high normalized pressure (*p* < 0.001), low normalized WSS (*p* < 0.001), and the center of divergent WSS vectors (*p* = 0.025).

There were some limitations to this study. This study included only 2 aneurysms with de novo bleb formation. Large-scale studies are needed to identify robust hemodynamic parameters related to de novo bleb formation and to subsequently predict aneurysms at a high risk of de novo bleb formation. Moreover, the validation of actual and virtual prebleb models is possible only when the results of CFD analysis for both models can be compared with those for models with 3D imaging data just before de novo bleb formation. It was extremely difficult to encounter a case that can offer 3D imaging data of unruptured aneurysms just before de novo bleb formation during the observation period. Accordingly, we validated the prebleb models by comparing the results of CFD analysis between actual and virtual prebleb models in the present study. Although the similarity in the results of CFD analysis for both actual and virtual prebleb models may be due to a small morphological change during the observation period, the larger change may have influenced the results of the CFD analysis. Although 3D digital rotational angiography is the gold standard to provide high-resolution images for CFD analysis, it is invasive and expensive. In this study, we used imaging data based on 3D CTA for CFD analysis. Generally, CFD analysis is based on several assumptions and approximations. It was assumed that the vessel walls were rigid; however, they are highly elastic structures and may deform during the cardiac cycle. Because the boundary condition for CFD analysis varies from patient to patient, it should be obtained by using phase contrast magnetic resonance imaging or transcranial Doppler ultrasonography for more precise evaluation of the hemodynamics in each aneurysm.

## Conclusion

De novo bleb formation on intracranial aneurysms may arise in areas associated with the combination of high normalized pressure, low normalized WSS, and the center of divergent WSS vectors. The multipoint method may be useful for statistical analysis of hemodynamics in a limited number of aneurysms. As a result of this study, we believe that we have found a more accurate indicator of de novo bleb formation than previous reports. If such a combination of hemodynamic factors is found in the aneurysm wall, it is expected to be prone to rupture and might be treated.

## Abbreviations

3D: Three-dimensional
AcomA: Anterior communicating artery
CFD: Computational fluid dynamics
CTA: Computed tomographic angiography
MCA: Middle cerebral artery
OSI: Oscillatory shear index
TAWSS: Time-averaged wall shear stress
WSS: Wall shear stress
